# Follow the leader: On the relationship between leadership and scholarly impact in international collaborations

**DOI:** 10.1371/journal.pone.0218309

**Published:** 2019-06-20

**Authors:** Zaida Chinchilla-Rodríguez, Cassidy R. Sugimoto, Vincent Larivière

**Affiliations:** 1 Instituto de Políticas y Bienes Públicos (IPP), Consejo Superior de Investigaciones Científicas (CSIC), Madrid, Spain; 2 School of Informatics and Computing, Indiana University Bloomington, Bloomington, Indiana, United States of America; 3 École de bibliothéconomie et des sciences de l’information, Université de Montréal, Montréal, Quebec, Canada; 4 Observatoire des Sciences et des Technologies (OST), Centre Interuniversitaire de Recherche sur la Science et la Technologie (CIRST), Université du Québec à Montréal, Montréal, Quebec, Canada; Max Planck Society, GERMANY

## Abstract

National contributions to science are influenced by a number of factors, including economic capacity, national scientific priorities, science policy, and institutional settings and cultures. Nations do not have equal opportunities to access the global scientific market, and therefore, often seek out international partners with complementary resources and expertise. This study aims at investigating national collaboration strategies, with a focus on research leadership—measured through corresponding authorship—and its relationship with scientific impact. Results show that countries with higher R&D investments are more scientifically independent, and confirm that international collaboration is positively related to citation impact. However, leadership in international collaboration is inversely related with a countries’ share of international collaboration and there is a very little relationship between citation impact and international leadership. For instance, most countries—and particularly those that have fewer resources—have higher scientific impact when they are not leading. This suggests that, despite increasing global participation in science, most international collaborations are asymmetrical, and that the research system remains structured around a few dominate nations.

## Introduction

In most social systems, there is a tension between cooperation and competition [1–2]. Scientific activity is no different: while researchers compete for the monopoly of scientific authority [3] they are also cooperating on collaborative projects [4–5]. The tension between cooperation and competition is also apparent in national science policies [6–7]. Scientific research can be seen as a strategic investment that can lead to competitive advantages in terms of economics, security, politics, and health [8]. National science agencies often tout their national competitiveness in production and impact. This emphasis on national production may be seen as antithetical to collaboration; however, the complexity and specialization of modern science has led to the internationalization of the research community [9–10], as evidenced through increased mobility and collaboration [11–19]. In the race for scientific impact, mobile researchers and internationally collaborative projects tend to lead to higher citations [20–26], exacerbating the cooperation-competition dynamic.

Research councils tend to encourage collaboration among different stakeholders as part of a funding strategy to maximize impact and stimulate economic growth [8, 27–28]. However, international collaboration can introduce additional challenges, as advanced and developing nations do not have the same access to the global scientific market [29]. Therefore, strategies of openness—which involve greater rates of collaboration and mobility [30]—have different costs and rewards for countries according to their capacity to trade researchers at various levels. This competitive agenda for science has been intensified by incentives to publish and the increasing focus on quantitative research evaluation, whereby publications are the major criterion for assessing researchers and institutions [31–34]. Collaboration is on the rise [1–2] and the increasing number of authors on a byline makes competition fierce for leadership positions (i.e., first, last, and corresponding authors), which signal dominant contributions to the scientific community. In order to understand and construct global indicators for science, we must first understand the role of these leadership positions in international collaborations.

This study analyses the leadership roles of nations, using corresponding authorship of internationally co-author publications as an indicator of leadership [35–46]. Specifically, we seek to answer the following research questions (RQ):

RQ 1: How do collaboration practices vary across countries?
RQ 2: How do advanced and developing countries vary in terms of their leadership roles in international collaborations?
RQ 3: What is the citation advantage of various collaboration strategies?
RQ 4: What is the citation advantage of leadership in international collaborations?
RQ 5: How do these relationships vary according to the scientific capacity of nations?

We consider three different types of papers (national, international, and non-collaboration), average normalized citations, as well as two indicators of scientific capacity: relative investments in R&D (GERD / GDP) and total numbers of papers. While the first indicator of research capacity is scale independent (i.e., relative), the second one is absolute and measures the ‘raw’ research capacity of a country. We analyze relationships and patterns in each country with a special emphasis on their significance when a country acts as a leader in international partnerships.

### Background

#### Scientific capacity and dependency

Government and industry R&D spending increasingly favors cooperation. Funding agencies, however, differ in their approach to collaboration. While European agencies foster international collaboration through funding programs [27], countries like the US tend to focus their funding internally, creating incentives for national collaboration [47]. These different approaches have implications for building and maintaining scientific capacity, defined as the infrastructure, investment, institutional and regulatory framework, and personnel available to conduct scientific research and technological development [48].

For developing countries, collaboration may have mixed benefits in building scientific capacity. There is increasing recognition of the need for greater efforts aimed specifically at building the capacity of developing countries to generate, disseminate, and use S&T to address both current and future needs in national, regional, and international arenas [49]. There remain, however, persistent disparities among countries in their capacity to create and use knowledge and technology for development and to participating and competing in the scientific and technology-based global marketplace [50–52].

Many countries with weaker scientific capacity depend upon international collaboration, which may impede the development of their capacity and diminish attention to topics of national priority [53–54]. Striking the balance between local and global science remains a challenge [55]. It has been debated whether international relationships fulfill the needs of developing countries: research topics may be more reflective of the research interests of international partners than those of their own country [47]. Therefore, relations of scientific co-operation among countries and processes of internationalization are understood as an unequal structure of output and divulgation of knowledge on the part of industrialized countries as opposed to peripheral ones [56–59]. Hence, while international collaboration is associated with higher scientific impact and economic growth, this relationship may not have symmetrical benefits [60]. This tension between national and international science is also reflected in research evaluation frameworks, in which publication-based evaluations create biases against the research agendas and dissemination languages of the non-English speaking countries [61–65].

#### Leadership in science

Leadership in scientific research has been the focus of several studies. These have shown that scientific leaders are associated with a capacity to recruit necessary resources and expertise to launch and sustain projects [48], and are associated with higher production and scientific impact [66–67]. From a bibliometric point of view, leadership has been measured through authorship position. Authorship is the mechanism through which researchers—and by extension the institutions, countries and geographical regions to which they belong—are acknowledged for their research activities and, thereby, demonstrate scientific capacity [32, 68–70]. The position of authors in the byline of scholarly publications can be determined by their contribution to a piece of research [33, 35, 71–73]. Despite disciplinary differences in authorship practices, we generally observe that first, last and corresponding author are more dominant contributors than middle authors [37, 69].

To limit irresponsible authorship listing, the International Committee of Medical Editors (ICMJE) decided on a number of authorship criteria that should be met, and details what the role of corresponding authors is. It states that the corresponding author takes primary responsibility for communication with the journal during the manuscript submission, peer review, and publication process, and typically ensures that all the journal’s administrative requirements are properly completed. The corresponding author should be available to respond to editorial queries in a timely way, and should be available after publication to respond to critiques of the work and cooperate with any requests from the journal for data or additional information should questions about the paper arise after publication [74].

Perception-based studies reinforce the dominant role of corresponding authorship [33, 37]. Being named as a corresponding author—generally the first or the last author [24, 31, 38, 40, 75–76]—confers greater acknowledgment, leadership, seniority or dominance; in contrast, absence in these roles could be associated with subordination or secondary role [43]. First and last-authored positions have also been used as proxies for leadership and indicators of the strength of a science system [73]. For example, the importance of author position—especially corresponding authorship—in promotion or tenure cases demonstrates the emphasis placed on these roles [32]. Some countries gone so far as to monetized this position of leadership: Korea, China, and Pakistan all have governmentally funded incentive structures for those who are first and corresponding authors on papers in journals such as *Science*, *Nature*, or *Cell* [77–78].

The concept of a research guarantor has also been suggested as an indicator of leadership. This concept considers the guarantor not to be an individual corresponding author, but rather the research group or institution to which the corresponding author belongs. Studies on guarantors have found differences in normalized impact and corresponding author distribution depending on the international collaboration rates and degree of scientific development of the collaborating countries [38]. The effect of the research guarantor on scientific impact was analyzed for more than 500 institutions worldwide demonstrating regional differences in the effects of leadership [39]. Analyses of the field of nanoscience and nanotechnology have shown that countries with the highest international collaboration present the lowest leadership [40]; opposite findings were obtained for Latin-American institutions, which have high leadership and low international collaboration in the field of Public Health and Medicine [42, 46]. Others found that developing countries’ scholarly impact is higher when they do not assume leading roles [42]. However, benefits in terms of citations may not be equally distributed among all countries engaging in these practices and may vary according to a leadership role and scientific capacities. For example, in the fields of tropical medicine, parasitology and pediatrics, countries with low and middle human development are less likely to lead international collaborations, and obtain much lower citation rates [43]. Some of these studies suggest that scientific collaboration and the establishment of alliances with more developed countries constitute an important mechanism through which less developed countries can be integrated into research activities. A review and validation of this approach has been recently published [44]. Building on these studies, in this analysis, we use corresponding authors’ country of affiliation as an indicator of scientific leadership in international collaboration.

## Materials and methods

Data for this paper were retrieved from Clarivate Analytics *Science Citation Index Expanded* (*SCIE*), *Social Sciences Citation Index* (*SSCI*), and *Arts and Humanities Citation Index* (*AHCI*). For the selected period (2000–2016), the database includes 19,460,980 papers (articles and reviews). The analysis is limited to the 94 countries that produced at least 7,000 documents over the entire period studied; those account for more than 98% of the world output (S1 Table). Research and Development (R&D) expenditures were drawn from the World Data Bank [79] for all countries except for Taiwan, for which we use OECD data.

For each of the countries analyzed, papers were grouped into three mutually exclusive categories, based on the institutional addresses of the authors: 1) papers that only have a single institution (no inter-institutional collaboration), 2) papers that have at least two institutions from at the same country (national collaboration), and 3) papers that have at least two institutions from at least two different countries (international collaboration). Leadership was measured through the country of affiliation of the corresponding author, and all others are considered as non-leading countries.

The number of citations of each paper was normalized by the average citations of all papers published in the same discipline in the same year [80–82], to obtain the Mean Normalized Citation Score. The field and subfield definition used here was that of the National Science Foundation. When the MNCS is above 1, it means that the papers have obtained, on average, impact above the world average; when it is below 1, it means the opposite. One of the focal points of the analysis is the degree to which a country benefits (as measured through citations) when it leads international collaborations. The benefit indicator is calculated as the difference between normalized citations when in a leading role versus a non-leading role, for internationally-collaborative publications. If the value is negative, the country does not derive benefits from collaborations when it is in a leading role. This difference should be interpreted within the contextual frame of the overall production of a given country.

Research and Development (R&D) expenditures as a proportion of GDP, defined by the World Bank, were used as an indicator of the economic capacity of countries. GERD is composed of three main components: Business Expenditure on R&D (BERD), Higher Education Expenditure on R&D (HERD), and Government Intramural Expenditure on R&D (GOVERD). We categorize countries into four groups: those countries investing more than 2% (17 countries—green color); those investing less than 2%, but more than 1% (17 countries—blue color); countries investing less than 1%, but more than 0.5% (18 countries—orange color); and countries investing less than 0.5% (40 countries—red color). As shown, these groups are not of equal size.

## Results

**Fig 1:**
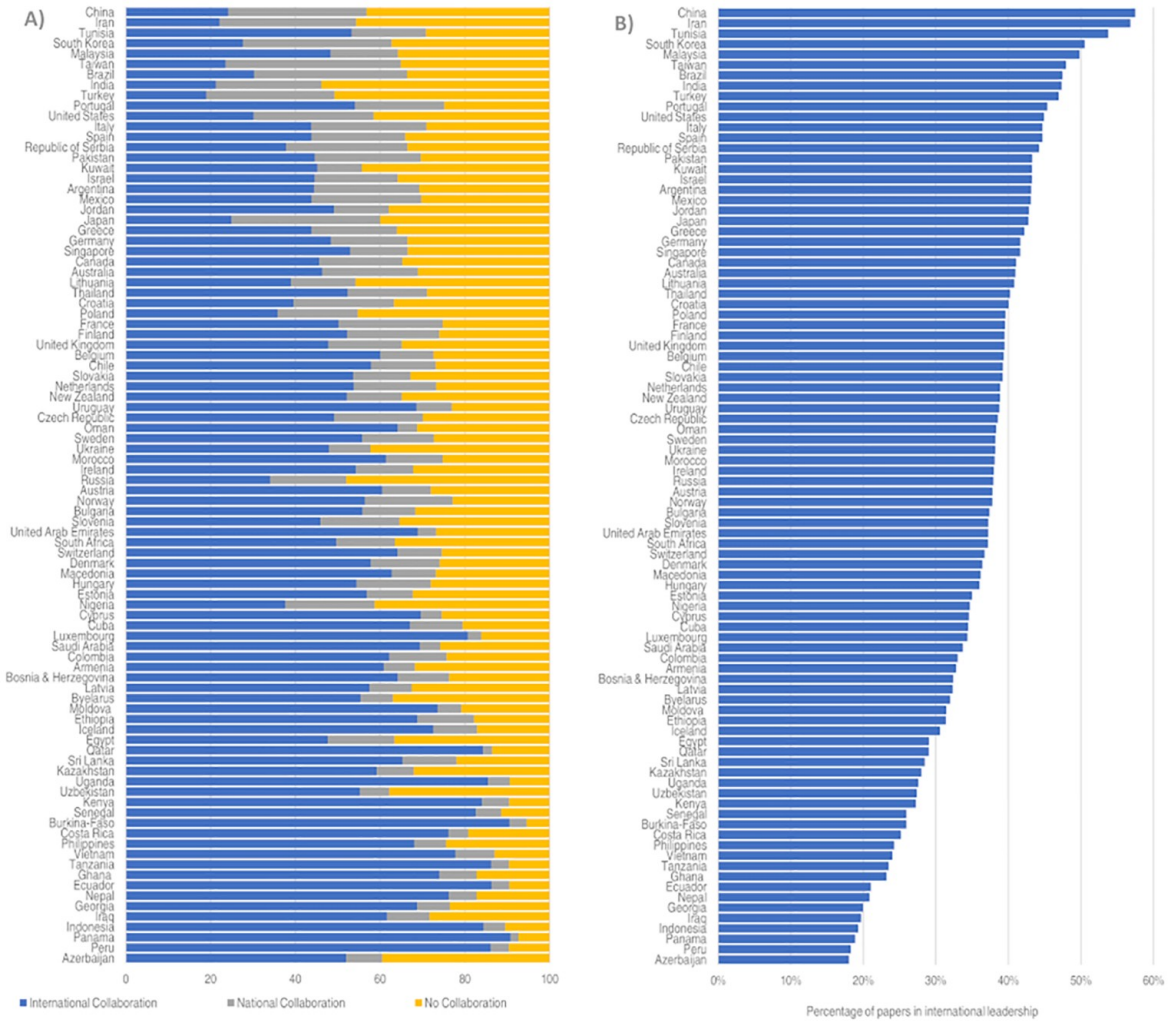
Percentage of papers in international collaboration, national collaboration and without collaboration (panel A), and proportion international collaboration papers in a leadership position (panel B), 2000–2016.

Countries vary in the proportion of their output that is a result of international collaboration (Fig 1). For many Asian countries (e.g., China, South Korea, Taiwan, and Japan), the proportion of domestic collaborations exceeds the proportion of international collaboration. On the other end of the spectrum, several smaller and less developed countries (e.g., Azerbaijan, Peru, Panama and Iraq, depend almost exclusively on international collaboration for their output, with low degrees of domestic collaboration and sole authorship.

These results are consistent with several previous results on collaboration, which demonstrate the effects of size, geographic proximity, and socio-political-economic affinities on the collaboration behavior of a country [83,84]. However, the situation is more nuanced when examining those countries with low degrees of international collaboration. While these countries—notably, China, Iran, Brazil, and the United States—vary in size and scientific capacity, they are each economic leaders in their region of the world, which might explain their lower proportion of international collaboration [17, 85]. Despite the differing opportunities for and engagement with international collaboration, an almost invariant inverse relationship can be observed between the percentage of international collaboration and the proportion of papers in which the country is in a leadership role. That is, the lower the international collaboration rate of a country, the more likely it is to serve as the lead on international collaborations. This reinforces the findings of previous studies [38,43–44].

These differences might be explained by differences in the scientific capacity of countries. We use number of papers as an indicator of scientific activity, and GERD/GDP of countries as an indicator of funding intensity. Taken together, these indicators provide a more informed understanding of underlying mechanisms of international collaboration and leadership.

**Fig 2:**
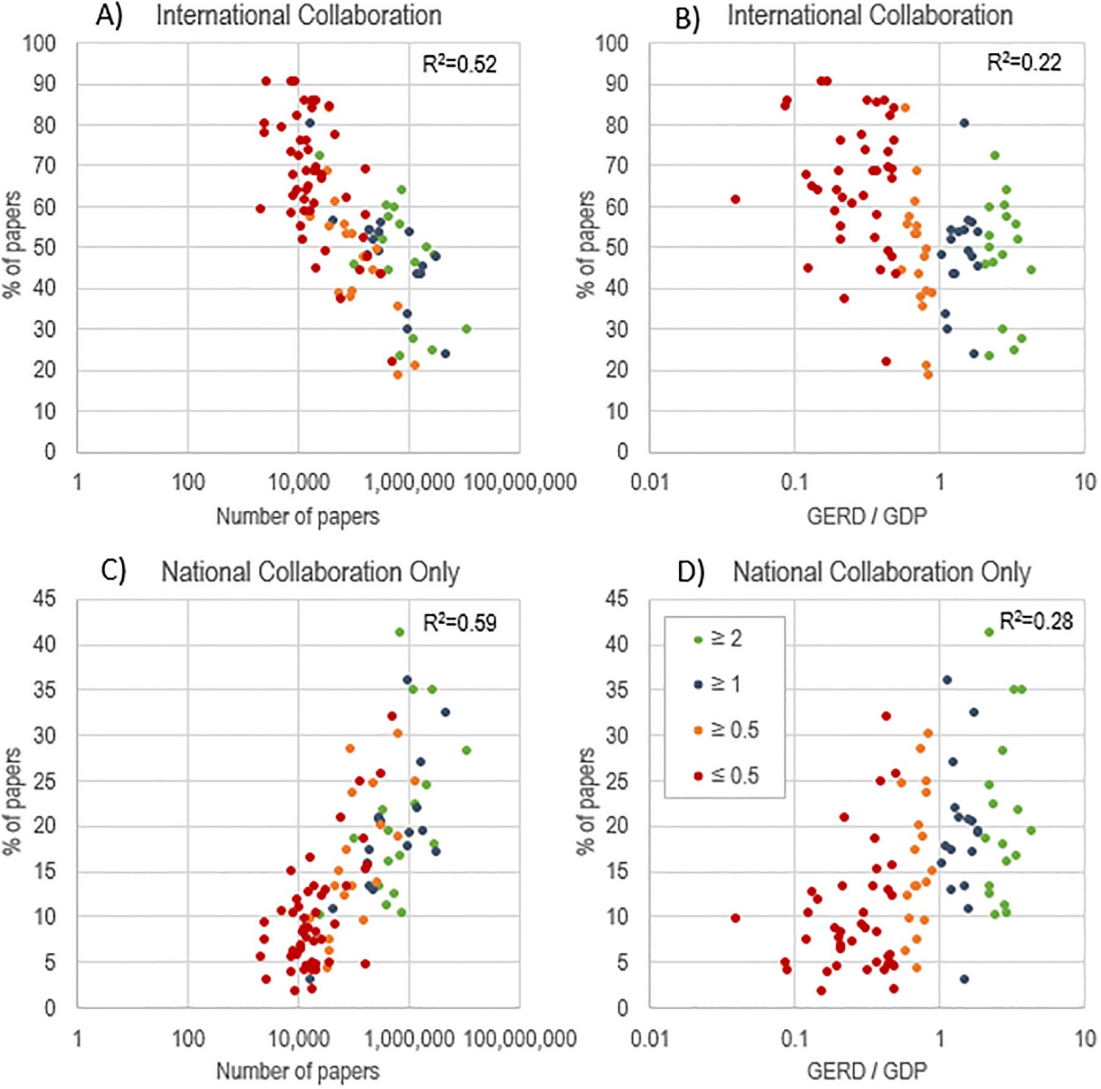
Relationship between the percentage of papers in international collaboration and number of papers (panel A) and GERD / GDP (panel B), and between the percentage of papers in national collaboration and number of papers (panel C) and GERD / GDP (panel D), by country, 2000–2016.

Fig 2 depicts the relationship between scientific capacity indicators (i.e., number of papers and GERD/GDP) and the proportion of papers in international and national collaboration (statistical analysis in Fig B in S1 Fig). As shown in the logarithmic plots, there is a negative relationship between the proportion of papers in international collaboration (with a significant coefficient of determination (0.52) and their GERD/GDP (0.22), whereas a positive relationship can be observed between the proportion of papers in national collaboration (0.59) and investment (0.28). Simply put, the greater the scientific capacity of a country, the more internalized the production. This is fairly intuitive: the more a country invests in R&D, the greater its capacity for creating infrastructure, training skilled researchers, attracting talent, and creating cohesion among domestic institutions. Countries with low investments are more dependent on resources and other forms of capital held by developed countries.

**Fig 3:**
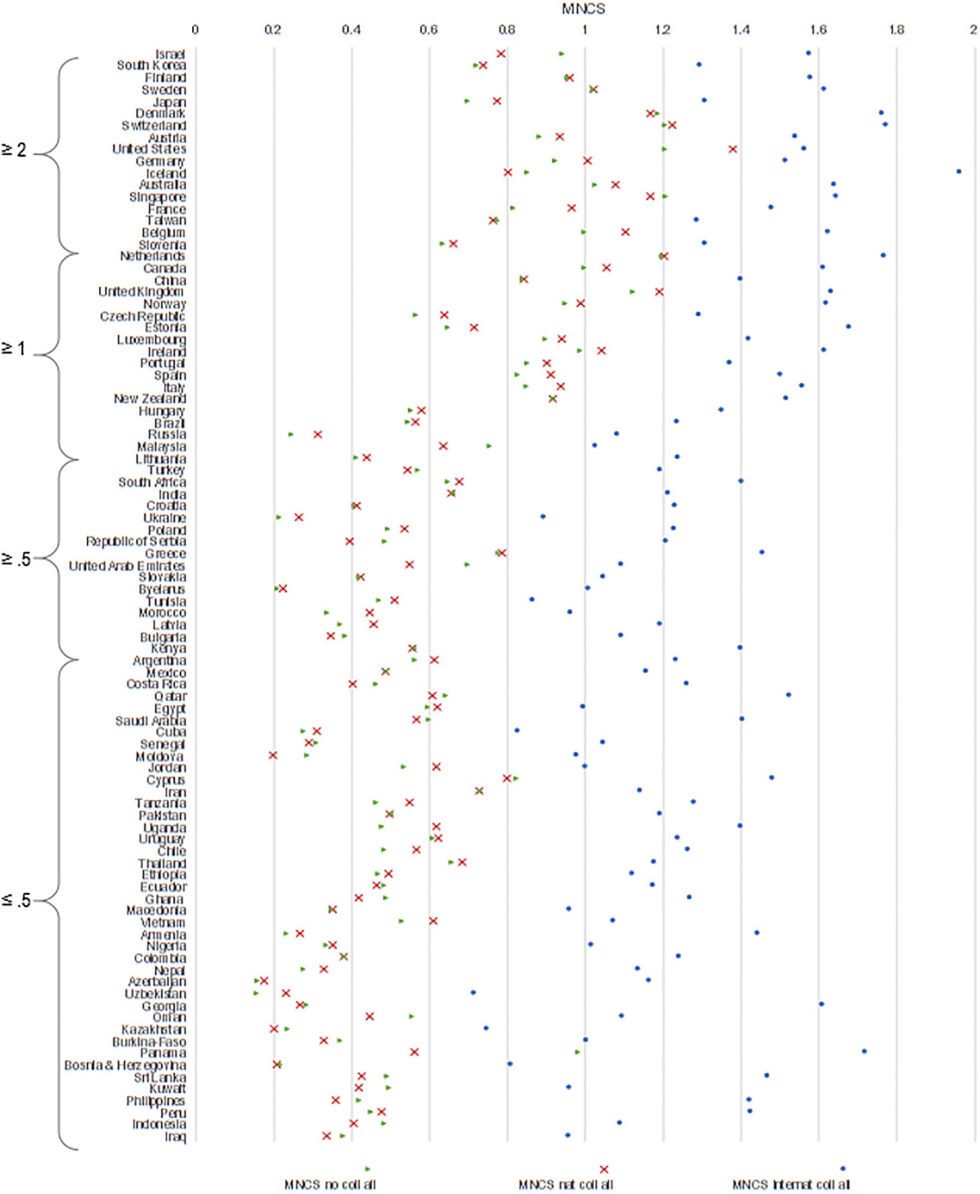
MNCS of papers with no collaboration, in national collaboration, and in international collaboration, by country, 2000–2016.

R&D investments are also strongly related to citation gains [30, 40, 86–87]. As shown in Fig 3, those countries that invest more than 2% of their GDP into R&D realize higher citation gains than those with lower levels of research investment, although the difference between papers in international collaboration and other papers is smaller. In all cases, international collaboration has a higher citation gain than national collaboration [88]. For countries with less than 1% of GDP dedicated to research, only the products of their international collaboration yield greater than the world average in terms of citations. On average, domestic output from these countries garners rates at lower than the world average. This suggests a strong relationship between international production and citation. The only countries which obtain citations above the world average in their non-collaborative leading outputs are Sweden, Denmark, Switzerland, the United States, Australia and Singapore.

**Fig 4:**
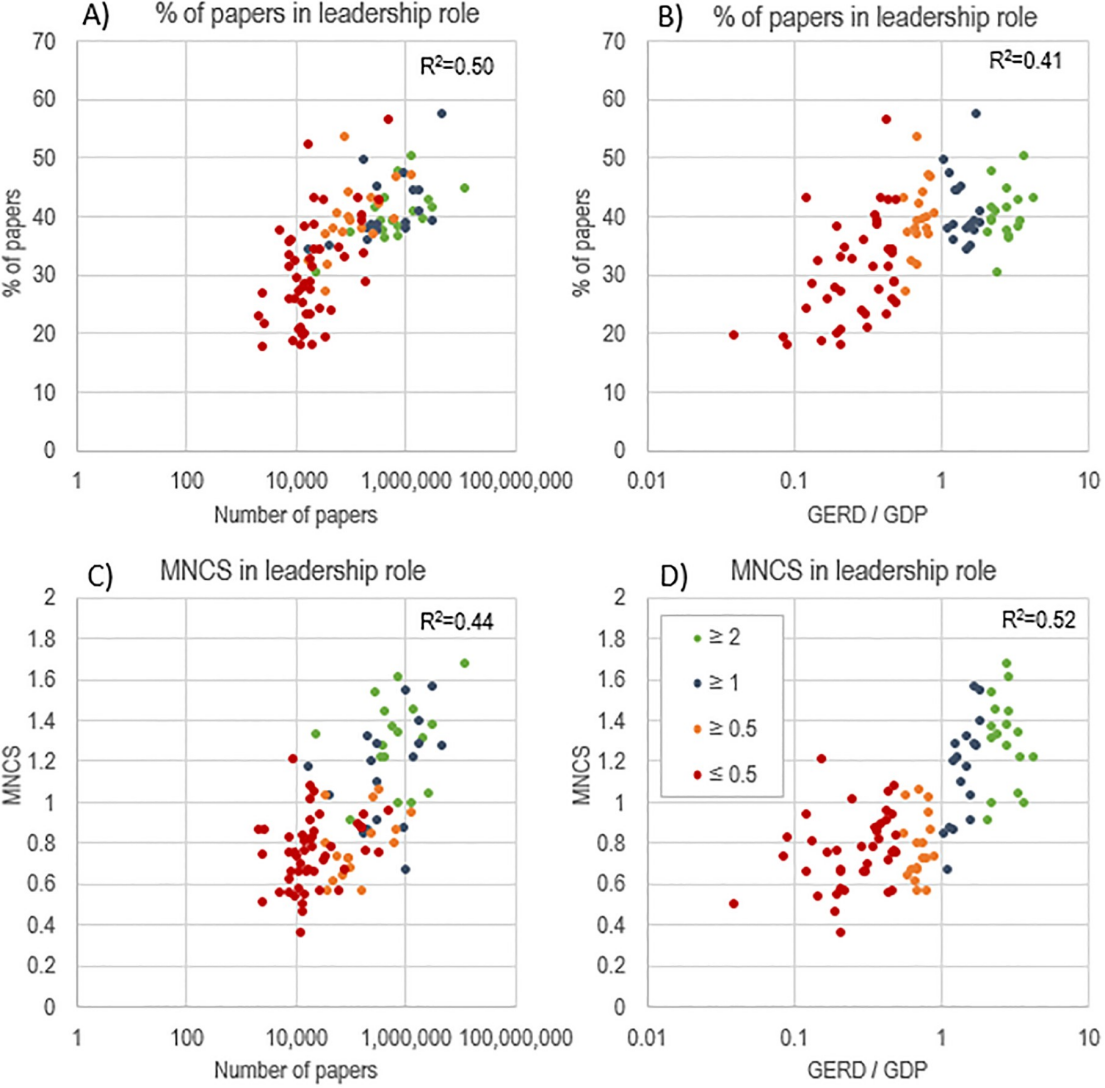
Percentage of papers in leadership role as a function of number of papers (panel A) and of GERD / GDP (panel B), and the MNCS in leadership role as a function of number of papers (panel C) and of GERD / GDP (panel D), by country, 2000–2016.

**Fig 5:**
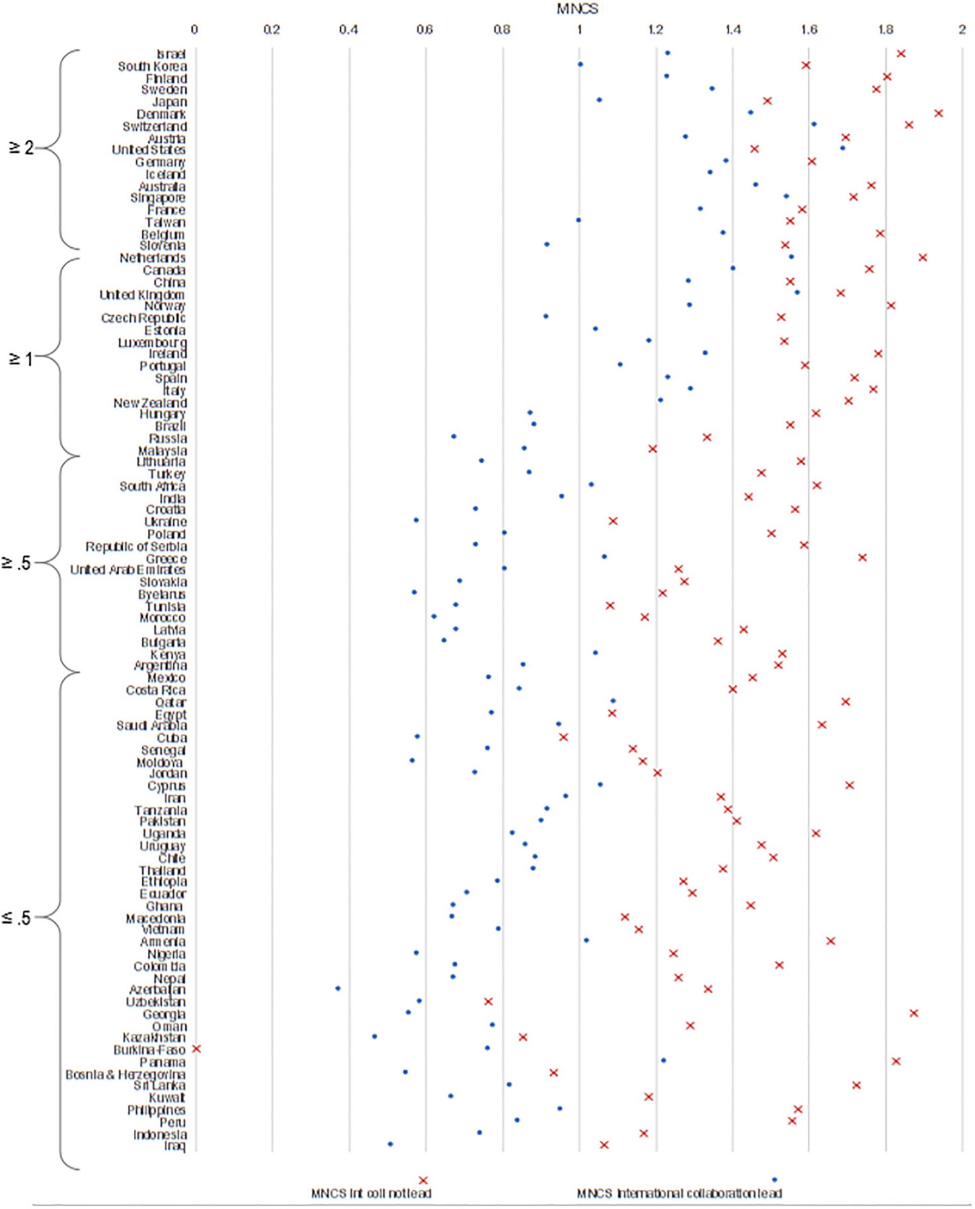
MNCS of papers in international collaboration, when in leadership role and when not leading, by country, 2000–2016.

Fig 4 presents the percentage of papers in leadership role as a function of number of papers (panel A) and of GERD / GDP (panel B), and the MNCS in leadership role as a function of number of papers (panel C) and of GERD / GDP (panel D). A logarithmic plot of the data shows that there is a significant coefficient of determination between the number of papers (0.5) and investment (0.41) with leading international papers (statistical analysis in in S2 Fig). Nations with greater research capacity—be it in terms of absolute numbers of papers or relative R&D investments—and research performance are more likely to serve as leaders and garner higher citations when they lead (Fig 5). Countries with the highest research investment obtain citation rates above the world average on any paper on which they collaborate—regardless of whether they are in a leadership position, with the exception of Slovenia (among the countries with the highest investment) and some Eastern European countries (i.e., Russia, Malaysia, and Turkey) among those investing more than 1%. However, countries with the lowest investment only achieve above world average positions when they are in non-leading positions (with few exceptions: i.e., Greece and Kenya).

**Fig 6:**
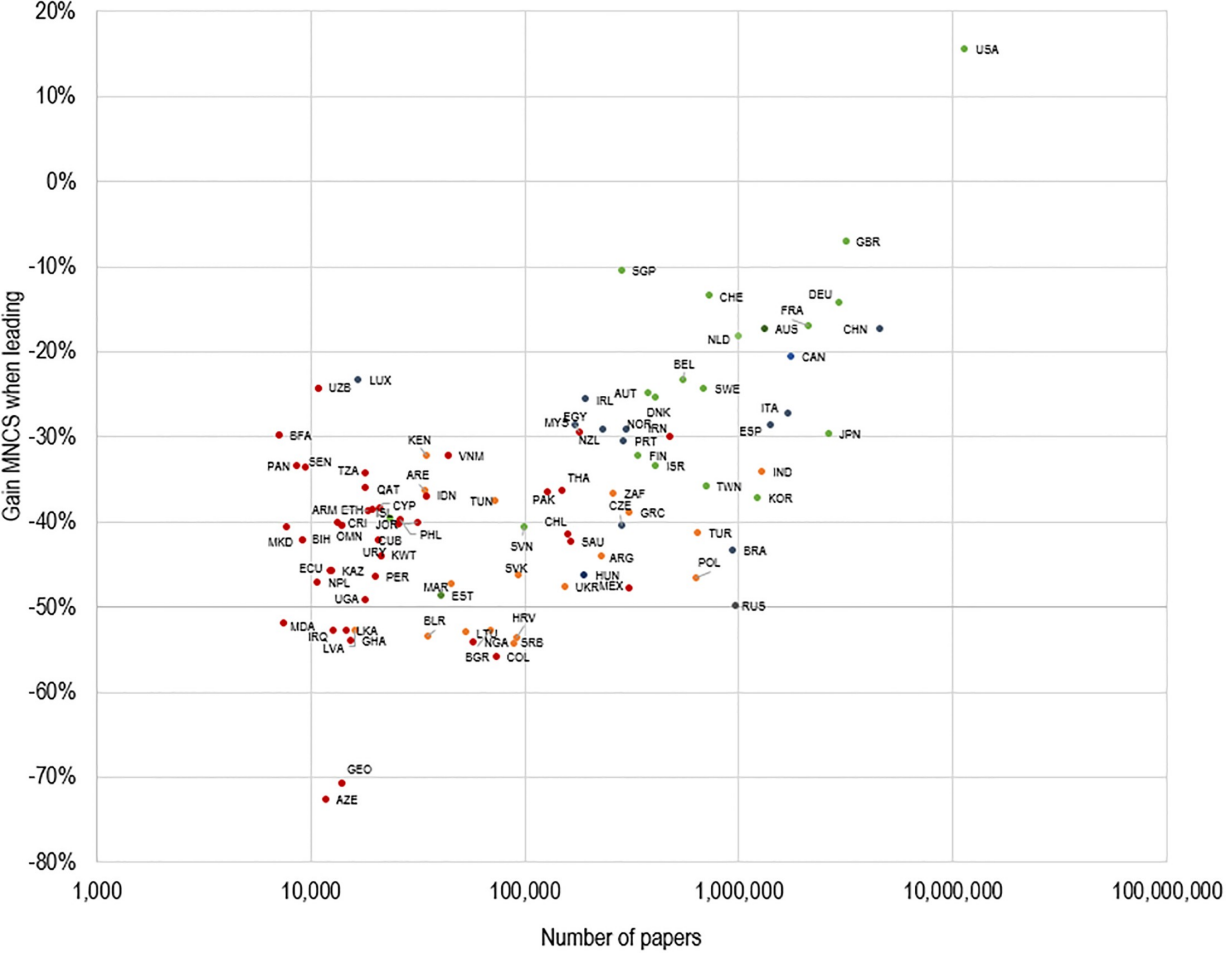
Gain in MNCS when leading international collaborations, by country, 2000–2016.

To investigate this further, we calculate the difference between the average field-normalized citation impact on papers when countries are in leading and non-leading positions (Fig 6). As shown, the United States is the only country that benefits, in terms of citations, when playing a leading role on scientific publications. In all other cases, there is a greater citation gain, on average, when countries assume a non-leading position. This corroborates findings of previous studies [38, 43–44].

**Fig 7:**
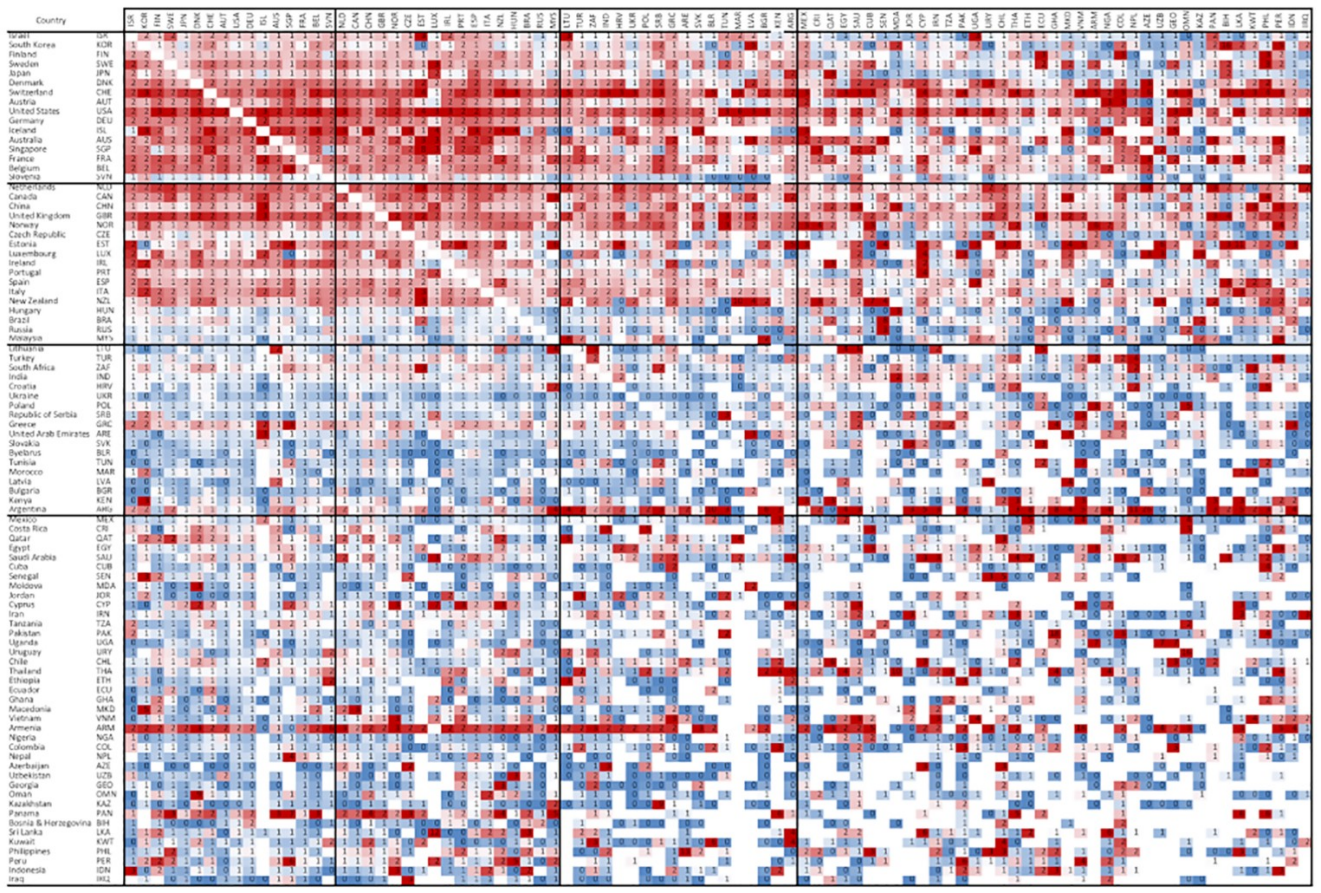
Matrix of the MNCS of international collaboration ties between countries, 2000–2016. Rows represent leading positions; columns represent non-leading positions. Results are presented as percentiles of MNCS.

This does not imply, however, that there is never a citation advantage for other countries when leading. This impact is often realized in localized partnerships or in partnerships of equalized research investment. Fig 7 presents a heat map of the MNCS of international partnerships by country, where the rows represent leading positions and the columns, non-leading positions. As evident, there is a relatively high citation payoff when countries with greater *relative* investment are in leadership positions. For example, Argentina achieves relatively high citation averages when leading with other countries of low scientific capacity.

## Discussion

Competition in the knowledge economy is affected by several factors: differences in institutional settings and cultures [5, 8], national scientific profiles [20, 89–90], scientific policies and economic capacities [47, 91], and mobility [26, 30], among other things. One avenue for overcoming these constraints is to seek out international partners with the necessary resources and expertise. Our analysis sought to understand the balance of this activity across nations and the relative advantages for countries according to their status as corresponding authors.

We found that the greater the research activity of a nation (in terms of number of papers), the more internalized the production. This is not a particularly novel finding: others have demonstrated that size often drives international collaboration [83–84, 92–93]. One should not, however, conflate size of country with size of production. For example, the United States and Japan both have highly productive scientific systems and similar profiles in terms of international collaboration. The higher the skilled personnel and amount of resources within a country, the more scientific independence; the inverse creates dependencies, as shown through the high rates of international partnerships [94].

These dependencies are not necessarily negative. Greater institutional cohesion among countries may have positive implications in nations’ performance and overall benefits for the geography of science [15, 39, 95–96]. These relationships are not politically neutral, however. For example, countries like Iran, Turkey, and India show the lowest level of internationalization (Fig 1), though the probabilities for establishing international partnerships are quite different among them. For example, Iran has experienced several embargos over the last decade and recently suffers visa restrictions from the US, which may affect their openness, whereas India and Turkey show high levels of international mobility [29]. Simply put, nations do not have the same opportunities to access to the global scientific market and the notion of openness works at different levels depending on the scientific capacities of countries and its ability to maintain and attract talent [30].

While international collaboration is positively related to citation impact (as shown in several other studies [20–22]), international leadership is inversely related to international collaboration and there is a very little relationship with citation impact and international leadership. As measured by MNCS, our results show that high levels of leadership without international collaboration may be an indicator of either research isolation (for small and developing countries) or consolidated scientific systems (for advanced and resourced countries). These findings are consistent with previous studies, which have shown that highly cited publications are negatively linked to leadership [39–40] since high values of leadership are more valuable and competitive for internationally collaborative papers [46].

Overall, nearly all countries have higher citation impact when they do not play a leading role in international publications. However, this gain differs considerably from one country to another (Fig 6). From a policy perspective, we might assume that countries with lower benefit rates (e.g., 20–25%) may be those with fairly high scientific capacity; whereas those with larger rates are those with strong dependencies. Only the most consolidated scientific systems, (e.g., United States, Switzerland, Sweden, Finland, Singapore and Australia), obtain higher citation impact when they lead international collaborations (with the exceptions of Greece and Kenya) (Fig 5). There is, of course, a tradeoff: even in the case of consolidated scientific systems, domestic collaboration directly increases national capacity, while foreign countries would potentially increase the knowledge base and resources available for local development.

### Limitations and future work

Research leadership based on corresponding authorship and international collaboration, although limited and not equally valid in all domains of science and scholarship [35–46], advances our ability to assign credit and responsibility at the global level. However, this is complicated by changing practices in authorship. Perhaps as a result of the emphasis on lead authorship in evaluation [97–98], there is a growing number of joint- first, last, and corresponding authorships [99].

It has been argued that analyses at lower levels of disciplinary aggregation more accurately represent the strengths of countries [20, 90]. This is certainly an element of analysis that requires further scrutiny—it can be hypothesized that concentration in certain specialties may have an impact on production and leadership. Additionally, as our analysis is based on mostly English language papers, a sizeable proportion of papers authored by researchers in non-Western countries are not covered, and those might exhibit different collaboration patterns [63–66].

Data on GERD is also imperfect. This is especially the case when grouped according to level of funding intensity, where there may exist important national disparities—particularly in the case of those countries with less than 0.5%. The use of this indicator instead of Government Budget Allocations or Outlays on R&D (GBARD) used in other studies [30, 87] is justified because we are interested in the level of infrastructure, human capital, and commitment in R&D activities for each country. Limiting the analysis only to government spending and/or Higher Education may be justified when analysis is focused in some specific nations [100], but that may exclude outputs and human capital funded by private companies.

Although indicators based on average citation counts are frequently used, they are also criticized in the literature [101–102]. Additional analyses should incorporate other indicators based on the idea of counting highly cited publications [103]. On the other hand, our analysis revealed the asymmetric relationship of citation impact and leadership (Fig 7): while there is a high degree of reciprocity in countries with high scientific capacity, the relationship becomes asymmetrical for countries with lower capacities. However, interesting regional variations can be observed, as well as unique outliers. These countries should serve as a focus for future study: understanding the mechanisms that make a country a high-impact regional leader will be an important consideration for policy purposes.

## Conclusion

Strategies for international collaboration do not equally benefit all countries. A disproportionately high reliance on international collaboration may imply that a country lacks the resources necessary to be independent. Collaboration may facilitate scientific advancement in that country, but it comes at a cost. For relatively disadvantaged countries, there is no citation benefit to lead research, but there is one for collaborating. In an era where bibliometric indicators play an important role in the allocation of resources, this may dissuade researchers and institutions from developing and leading their own research agenda, with potentially negative consequences in terms of linguistic and topical diversity. Robust science policy must take into account inequalities in labor and reward rather than defaulting to a universal strategy towards international collaboration.

## Supporting information

S1 Fig Results of statistical analysis describing relationship between scientific capacity indicators and the proportion of papers in international and national collaboration. (XLSX)

S2 Fig Results of statistical analysis describing relationship between the scientific capacity indicators and the proportion of leading international papers. (XLSX)

S1 Table Data and indicators used in the study. (XLSX)
